# A New Skin Tensiometer Device: Computational Analyses To Understand Biodynamic Excisional Skin Tension Lines

**DOI:** 10.1038/srep30117

**Published:** 2016-07-25

**Authors:** Sharad P. Paul, Justin Matulich, Nick Charlton

**Affiliations:** 1 Dept. of Skin Cancer, School of medicine, University of Queensland, Brisbane, Australia; 2 Faculty of Surgery, University of Auckland, Auckland, New Zealand; 3 Auckland University of Technology (AUT) 55 Wellesley St E, Auckland 1010, New Zealand; 4 Skin Surgery Clinic, 271 A Blockhouse Bay Rd, Auckland 0600 New Zealand; 5 Electrical and Electronic Engineering Department, Auckland University of Technology (AUT), 55 Wellesley St E, Auckland 1010, New Zealand; 6 Industrial Design & Innovation, Auckland University of Technology (AUT) 55 Wellesley St E, Auckland 1010, New Zealand

## Abstract

One of the problems in planning cutaneous surgery is that human skin is anisotropic, or directionally dependent. Indeed, skin tension varies between individuals and at different body sites. Many a surgeon has tried to design different devices to measure skin tension to help plan excisional surgery, or to understand wound healing. However, many of the devices have been beset with problems due to many confounding variables - differences in technical ability, material (sutures) used and variability between different users. We describe the development of a new skin tensiometer that overcomes many historical technical issues. A new skin tension measuring device is presented here. It was designed to be less user-dependent, more reliable and usable on different bodily sites. The design and computational optimizations are discussed. Our skin tensiometer has helped understand the differences between incisional and excisional skin lines. Langer, who pioneered the concept of skin tension lines, created incisional lines that differ from lines caused by forces that need to be overcome when large wounds are closed surgically (excisional tension). The use of this innovative device has led to understanding of skin biomechanics and best excisional skin tension (BEST) lines.

Understanding the tensile strength of wounds is critical in planning surgical techniques^1^ and understanding wound healing^2^. The concept of skin tension lines is widely attributed to the studies of Langer, who used a round-tipped awl to create defects in cadaveric skin and then observed the elongation of these circular defects due to the underlying wound tension^3^. Langer perhaps never intended these lines as surgical excisional lines, even though surgeons all over the world began adopted his diagrams while planning surgical procedures. Later, Borges, while studying wrinkle lines and their applications in plastic surgery, introduced the concept of skin relaxation rather than skin tension i.e. he felt skin tension rays would radiate in all directions, except one and that would be the relaxed skin tension line (RSTL). He advocated planning excisions using such lines^4^.

When it comes to measuring wound tension, there are generally two methods –Harvey’s technique measuring intraluminal pressure inside a hollow organ^5^ or that advocated by Howe – studying the forces needed to disrupt a wound^6^.

Many different tensiometers have been developed in studies to measure wound tension^6^,^7^ but most have been cumbersome, non-portable and use clamps – the placement of which becomes very user-dependent. Thompson and others reported an improved design of a tensiometer to study irradiated wounds in rabbits, but images of their device show a large non-portable device^8^. Spring loaded sensors have also been developed to measure the force on a tensioned suture inside a closed incision and to measure the pulling force used to close the incision^9^ – however, once again results are variable due to variability of knot-tying techniques of sutures used.

Jacquet^10^ and others have suggested that when it comes to skin there is a difference on stress-strain relationships *in vivo* and *ex vivo* measurements – if one observed the shape of the *ex vivo* test, the stress-strain curve starts with a very low slope and then presents a large non-linearity at low strain, whereas in an *in vivo* test, the curve is much stiffer. This stiffness is attributed to the initial stress of the skin, also noted by Langer. Therefore Jacquet’s team designed a skin tension measuring device that relied on extensiometry testing i.e. skin was tested not only in traction, but also in compression^10^. In our view this device as still not portable or user-friendly enough to be used in curved or smaller bodily sites during real-time surgery.

Our planning brief was therefore simple for this design project. Could we come up with a bi-directional skin tension measuring device that could measure inherent tension in the skin, and also the force needed to close the wound i.e. one that could measure inward and outward forces, while having the ease of use of a forceps? How could we achieve consistency of results, while reducing the variability of readings?

## Materials and Methods

**Figure 1:**
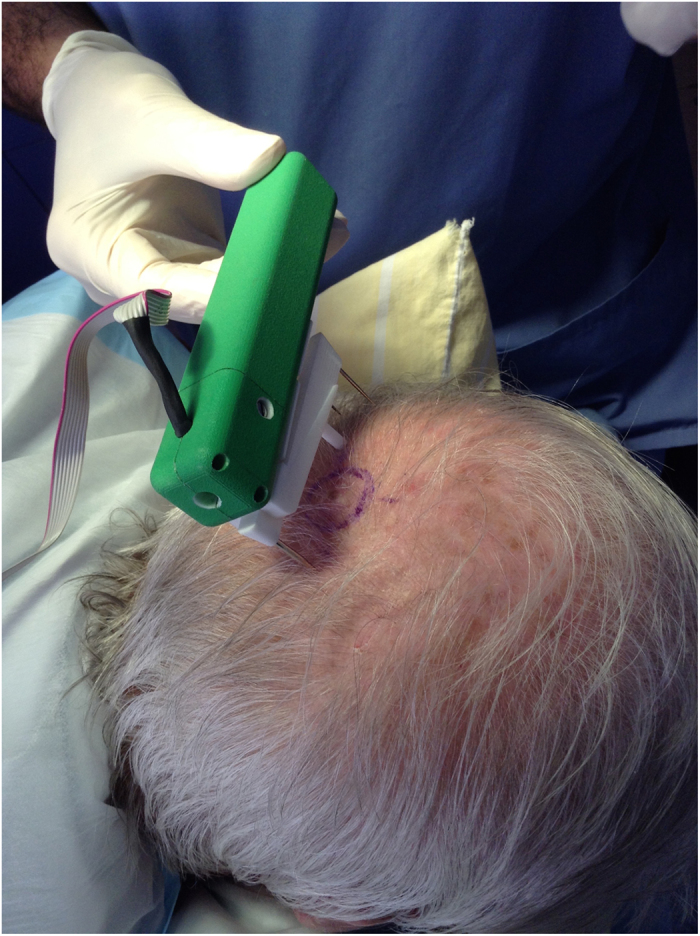
Skin tensiometer being used to measure scalp tension.

The inspiration and influence behind the current design and style (Fig. 1): The current device is designed to be used ‘remotely’, in other words the user is only required to operate the controls, and not actually be in contact with the measuring portion of the device. This particular style was developed after several reviews and iterations of hand held devices that proved to be ineffective, and provided nonsensical data resulting in inaccurate readings and inconstant results.

**Figure 2:**
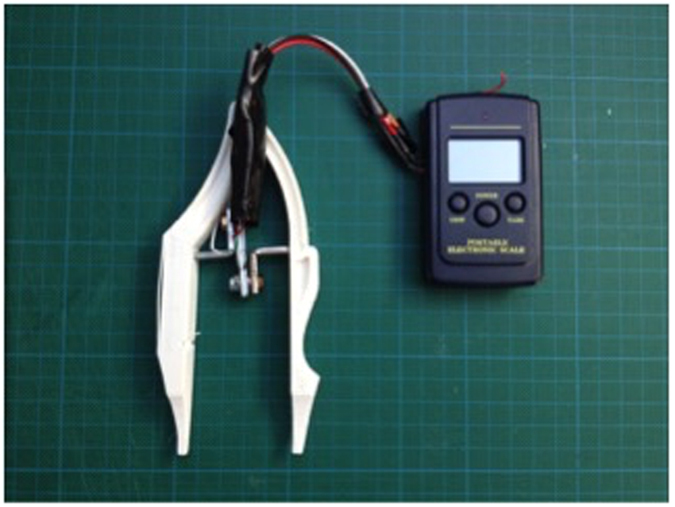
Initial forceps-like design.

The very first prototype we designed was based around the idea of the device operating like a pair of forceps, using the device to stretch or compress the skin, whilst using flex sensors on the arms of the device to calculate the resulting tension on the skin (Fig. 2). However, during the development stages several potential issues were discovered, as to obtain consistent results the user had to apply a consistent and constant force each time a measurement was taken. Also while using the device any instability’s in the users hand caused fluctuations in the readings.

Due to the problems described above it became clear this device would need to be able to take measurements automatically without direct user support (other than operating switches). The device was also designed to be bi-directional i.e. the user can measure inward and outward forces by flicking the switch to change the direction of measurement. This allows us to measure any inherent skin tension (pre-tension) and understand both skin tension and relaxation lines.

The current device is made up of four main elements, a linear actuator, a force sensor, signal conditioning hardware and embedded software:

1. The linear actuator provides the consistent force that is applied to the skin in order to measure the resulting tension. The use of a linear actuator means if the device is securely fastened and the prongs are attached correctly then for each measurement the applied forces are consistent and repeatable. The linear actuator position is set using a varying-pulse-width waveform that is driven by a microcontroller in the control box.
2. The force sensor is a strain element force sensor with a sensing range of 0–10 newton’s (N). The reactive force applied by the skin acts via a pivoting arm on the force sensor. The force is converted to a proportional voltage potential, which is put through signal conditioning and converted in software into a force to be shown on the display.
3. The signal conditioning hardware is made up of an instrumentation amplifier, which takes the tiny potential difference across the force sensor, and amplifies it to a usable voltage. On the output stage of the amplifier is a low pass RC filter circuit with an upper cut off frequency of 40 Hz, this is used to remove high frequency noise introduced from vibrations and external interference such as mains hum.
4. The embedded software has three tasks, it provides the control for the linear actuator, it converts the conditioned signal voltage from the force sensor into a measured tension, and it drives the display where the measurements are displayed.

To take a tension measurement the software starts by taking a calibration reading, this reading is known as the zero tension point. The software then instructs the actuator to stretch the skin a predefined distance, the software then takes another reading which is known as the final tension point. The software then instructs the actuator to return to its previous position releasing the tension on the skin. The final step is for the software to display the resulting tension which is the difference between the zero tension point and the final tension point (Tension = abs (ZeroTensionPoint – FinalTensionPoint)).

The algorithm to calculate the tension ultimately is the difference between two force measurements as described above; these force measurements are derived from a digital ADC reading (0–1023) which is converted to grams using the formula (g = ADC value * 0.25). We were then able to convert this back into Newtons (N) for standardization and publication of results.

**Figure 3:**
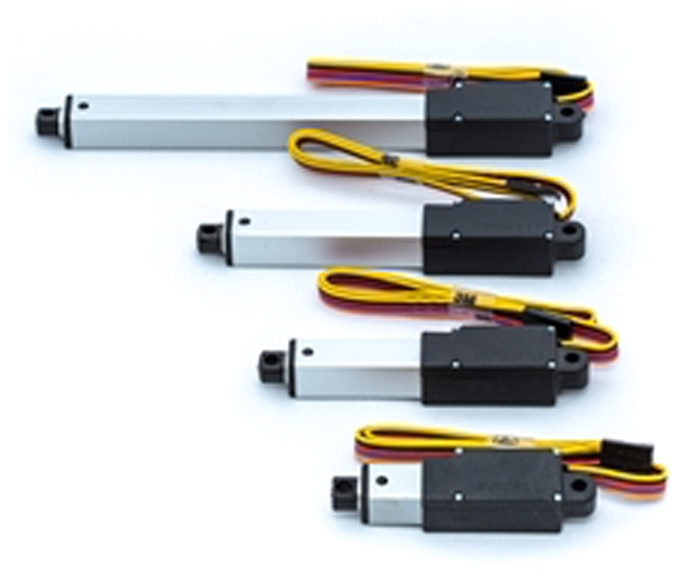
Linear actuator.

The linear actuator used was a L12-P Micro Linear Actuator with Feedback (Firgelli Technologies Inc. BC, Canada) – this device (Fig. 3) has an internal potentiometer that can be used to provide position feedback. However, it does not have any internal controller or limit switches.

**Figure 4:**
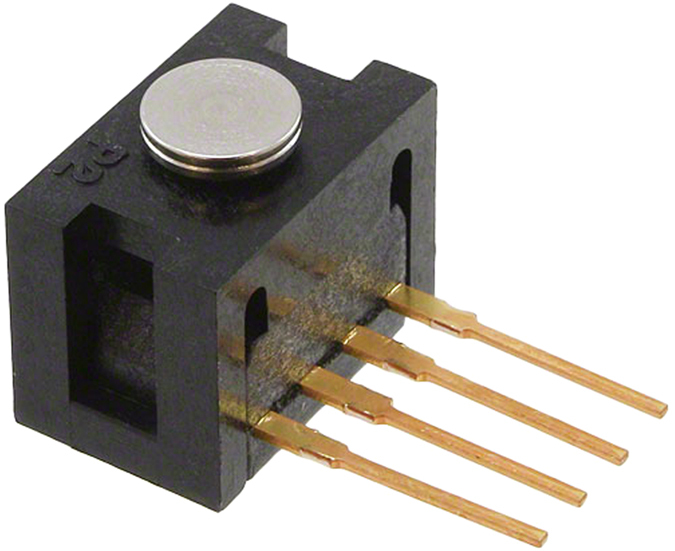
Force sensor.

Force Sensors used were Honeywell (Honeywell Corporation, USA) Force Sensors (Fig. 4) with a sensing range of 0–10 N. We chose this sensor and sensing range as previous researchers have already done preliminary work to establish the range of forces encountered in human wounds and pigskin.

The methods were carried out in accordance with the approved guidelines and all experimental protocols were approved by a named institutional and/or licensing committee –Ethics ref: 15/CEN/113 Health and Disability Ethics Committee, NZ and Institutional Human Research Ethics Approval 2015001550, University of Queensland (ethics approvals were obtained for this study from the relevant authorities in both New Zealand and Australia, given the lead author’s academic affiliations). Informed consent was obtained from all human subjects. There were no animals specially obtained or killed for or during this study.

We first began by studying pigskin, and later scalp skin to ascertain the kind of tension forces we would be dealing with. In human scalp wounds, it has been reported that the tension needed to approximate skin without any tension reduction suture (S) can be as high as 6.5 ± 4.6 N (Newtons)^11^. Other researchers, who tested human skin over the trunk concluded that the maximal force varied from 0.5 N for the small wounds (30 * 5 mm) to 1.5 N for the medium-sized wounds (30 * 15 mm), to a peak force of 3.2 N for large (46 * 13 mm) wounds^12^. Authors studying pig skin by creating massive elliptical defects (8 cm * 5 cm) reported that the mean force needed to close non- undermined surgical wounds was 15.7 N compared with 11.5 N for undermined wounds an the animals^13^. Given we set out to investigate skin tension lines after excisional surgery for skin cancer, we determined that a range of 0–10 N was adequate for our purposes. In our testing on scalp wounds, we found a range of tension measurements from 0.5 N to 4.6 N.

The software, in our case, was written in C by Justin Matulich, the member of our team from the electronics and electrical engineering department at the Auckland University of Technology (AUT). The micro controller used was the ATMEGA32 (Atmel Corporation, USA) – a low-power CMOS 8-bit microcontroller based on the AVR enhanced RISC architecture. The ATmega32 achieves throughputs approaching 1 MIPS per MHz allowing our system designer to optimize power consumption and processing speeds.

## Discussion

**Figure 5:**
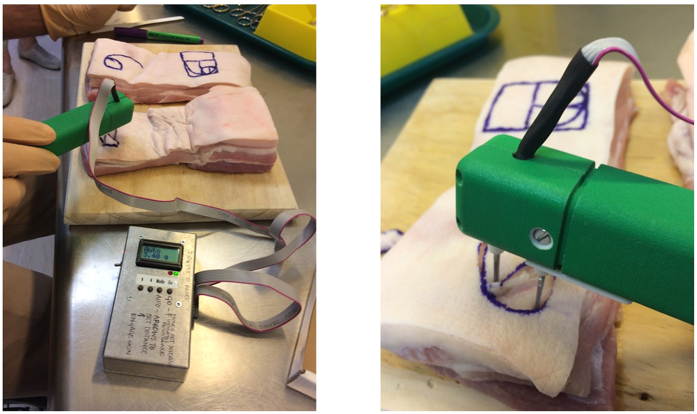
Comparison of tensions resulting in closures using different types of surgical skin flaps on pig skin.

We developed an initial prototype that was tested in pigskin (Fig. 5) and optimized and calibrated to achieve consistent results before our latest design with autoclave-able tips for human skin (Fig. 1). In pigskin we used our device to test wound tension for primary elliptical surgical closures and also to compare and contrast different cutaneous flap techniques, the results of which form the basis of a different surgical paper. On the scalp, the device allowed us a great understanding of the biodynamics of previously noted Langer’s Kraissl’s and Borges’ lines and determine the best methods of closing scalp defects. Fundamentally, we were able to determine the biomechanical differences between ‘tension’ and ‘relaxation’ skin lines and determine the best lines of closure for excisional wounds. Again, these detailed findings will be published in a surgical journal as this article is to present our new innovative tensiometer, that is a major improvement on previous devices of this nature.

In conclusion, the device presented in this paper is a major improvement on previous models due to the following:

1. It is relatively user-independent. In our final design, we incorporated a bracing bar that steadies the device and negates any operational hand tremors. However, it does take some understanding and practice to use the device and achieve completely reliable results. In our case, the lead author has now been able to use the device in different settings and achieve consistent results.
2. The system is relatively portable and the ability to measure skin tension at any zone and body site is a major advance over previous devices.
3. Having obtained reliable results and important findings re skin tension for scalp wounds, the author is continuing this study in other anatomical locations and these will be published in further papers, as the findings become available.

## Additional Information

**How to cite this article**: Paul, S. P. *et al.* A New Skin Tensiometer Device: Computational Analyses To Understand Biodynamic Excisional Skin Tension Lines. *Sci. Rep.* **6**, 30117; doi: 10.1038/srep30117 (2016).
